# Preparation and Characterization of Bio-Based PLA/PEG/g-C_3_N_4_ Low-Temperature Composite Phase Change Energy Storage Materials

**DOI:** 10.3390/polym15132872

**Published:** 2023-06-29

**Authors:** Liu Feng, Junjie Ding, Hengming Hu, Zichun Lv, Yongsheng Zhang, Boqiang Xu, Jingru Quan, Shijie Hao, Haojie Fan, Zusheng Hang

**Affiliations:** 1 School of Materials Science and Engineering, Nanjing Institute of Technology, Nanjing 211167, China; f2928912158@163.com (L.F.);; 2 School of Chemistry and Chemical Engineering, Nanjing University of Science and Technology, Nanjing 210094, China

**Keywords:** polylactic acid (PLA), biodegradable, composite phase change energy storage material (CPCM), torque rheometer mixing

## Abstract

As energy and environmental issues become more prominent, people must find sustainable, green development paths. Bio-based polymeric phase change energy storage materials provide solutions to cope with these problems. Therefore, in this paper, a fully degradable polyethylene glycol (PEG20000)/polylactic acid (PLA)/g-C_3_N_4_ composite phase change energy storage material (CPCM) was obtained by confinement. The CPCM was characterized by FTIR and SEM for compatibility, XRD and nanoindentation for mechanical properties and DSC, LFA, and TG for thermal properties. The results showed that the CPCM was physical co-mingling; when PLA: PEG: g-C_3_N_4_ was 6:3:1, the consistency was good. PEG destroys the crystallization of PLA and causes the hardness to decrease. When PLA: PEG: g-C_3_N_4_ was 6: 3: 1, it had a maximum hardness of 0.137 GPa. The CPCM had a high latent enthalpy, and endothermic and exothermic enthalpies of 106.1 kJ/kg and 80.05 kJ/kg for the PLA: PEG: g-C_3_N_4_ of 3: 6: 1. The CPCM showed an increased thermal conductivity compared to PLA, reaching 0.30 W/(m·K),0.32 W/(m·K) when PLA: PEG: g-C_3_N_4_ was 6: 3: 1 and when PLA: PEG: g-C_3_N_4_ was 3: 6: 1, respectively. Additionally, the CPCM was stable within 250 °C, indicating a wide appliable temperature range. The CPCM can be applied to solar thermal power generation, transportation, and building construction.

## 1. Introduction

With the increasing prominence of energy and environmental issues, the demand for energy conservation and environmental protection has been continuously increasing. Therefore, while new energy is developing rapidly, people must also consider environmental issues and seek a sustainable, green development path [1,2]. Low-temperature phase change energy storage materials have applications in fields such as solar thermal power generation, transportation, thermal energy management [3], waste heat recovery [4], and building energy conservation [5]. In the process of developing phase change energy storage materials (PCMs), researchers have studied many different types of phase change materials, including inorganic compounds (such as salts, hydrates) and organic compounds [6] (such as paraffin, fatty acids), as well as polymeric materials (such as PEG). Polymeric phase change materials have the advantages of a high energy storage density, good thermal conductivity, good chemical and cycling stability, low toxicity and low corrosion. Moreover, such materials are easy to design and regulate in terms of their structures and are thus easily processed and formed in practice. Tang et al. [7] found that the enthalpy of SAL/HDPE/EG composite materials can reach 200 kJ/kg, which is much higher than that of ordinary energy storage materials such as paraffin. However, its thermal conductivity is poor, only 0.20 W/(m·K). Therefore, Tang et al. [8] studied MA/HDPE composite materials to improve the thermal conductivity to 0.45 W/(m·K). In summary, polymeric phase change materials can perform excellent energy storage functions. However, polymeric materials have a limited service life, and aging can cause changes in their properties. Improper handling after aging can also cause environmental pollution [9].

Bio-based materials provide one option to address these issues [10,11]. Polyethylene glycol (PEG) is a degradable material and can serve as a high-quality energy storage material, but its flexibility is too high to be used as a structural material [12]. Polylactic acid (PLA) is the most widely used biodegradable material with high strength. It can decompose into carbon dioxide and water in the environment without causing pollution and has some unique characteristics for energy storage. Firstly, PLA has a relatively high enthalpy value [13]. Secondly, the high crystallinity of PLA gives it good heat resistance at low temperatures (melting point of 165–180 °C), which can be applied to phase change energy storage. However, the thermal conductivity of PLA is not high [14], between 0.1–0.3 W/(m·K), which obviously needs to be addressed. Currently, phase change energy storage materials are usually compounded with carbon black to enhance their thermal conductivity. However, carbon black can only be made into darker-colored energy storage materials, which limits its application field, and carbon black is not flame-retardant. Graphitic carbon nitride (g-C_3_N_4_) nanoparticles [15], with a thermal conductivity of 45.4 W/(m·K) [16], were used as thermally conductive enhancers in this study, which have an excellent porous structure [17], thermal stability, a rich variety of nano-multilevel structures, are rich in N, and have flame-retardant properties [18].

In summary, in this paper, PEG and PLA were blended with the thermally conductive enhancer g-C_3_N_4_ using a torque rheometer [19] to prepare the composite phase change energy storage materials (CPCMs) [20]. The samples were processed into thin cakes using a flat vulcanizing machine. The CPCMs remained solid during the phase change process. This type of polymer solid–solid phase change material was prepared by physical blending methods, which combine solid–liquid phase change materials with polymer matrices through physical actions (such as intermolecular forces or hydrogen bonding) or encapsulation techniques, so that the phase change materials lose their fluidity macroscopically, thus preparing composite solid–solid phase change materials. In addition, phase change energy storage mainly stores heat, which essentially relies on the latent heat of the phase change during the phase change process to control the absorption and release of energy, thereby achieving the purpose of thermal energy storage and temperature regulation, and improving the utilization of energy.

## 2. Materials and Methods

### 2.1. Materials

Polyethylene glycol (PEG20000, ≥99.0%) was purchased from BASF China Co., Ltd. (Shanghai, China) as white, wax-like flakes. Polylactic acid (PLA, ≥99.0%) was purchased from Zhejiang Hisun Biomaterials Co., Ltd. with the product name of REVODE290, and received in the form of pellets. Melamine was purchased from National Pharmaceutical Group Chemical Reagent Co., Ltd. (Shanghai, China).

### 2.2. Formulation and Preparation of CPCMs

#### 2.2.1. Preparation of g-C_3_N_4_

**Figure 1:**
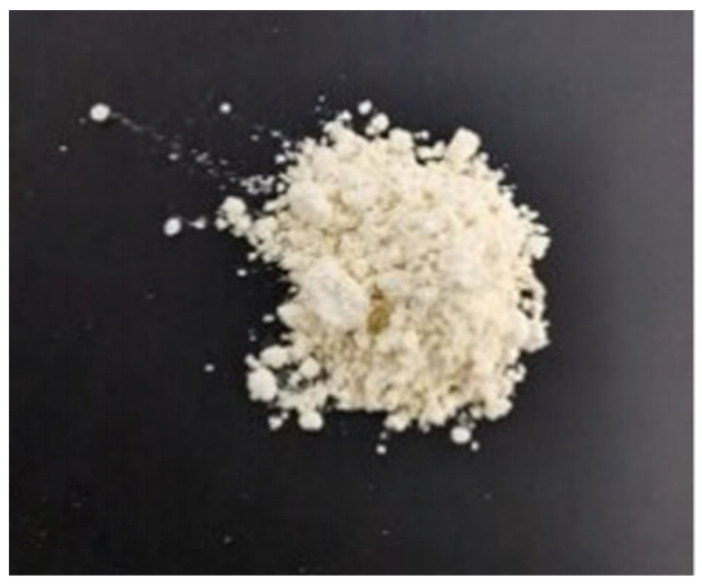
g-C_3_N_4_ powder.

Melamine was placed in a crucible and fully calcined at 550 °C in a muffle furnace for 3 h, obtaining a mixture of light-yellow g-C_3_N_4_ and white melamine. The mixture was washed with alcohol three times and distilled water three times, then ground to fine and uniform particles using an agate mortar, and finally dried in a 60 °C oven for 2 h, obtaining light yellow g-C_3_N_4_ powder [21], as shown in Figure 1.

#### 2.2.2. Formulation of CPCMs

The formulations of the composite phase change energy storage materials (CPCMs) are shown in Table 1. Numerous studies have been conducted on the blending of low molecular weights and low contents of PLA and PEG. In this study, high molecular weights (200,000) and high contents (greater than 30 wt.%) of PEG were blended with PLA and g-C_3_N_4_ as a thermal conductive enhancer to prepare CPCMs [22]. Sample A1 was pure PLA, sample A2 was pure PEG20000, samples A3–A5 had 10 wt.% g-C_3_N_4_, with sample A3 containing 30 wt.% PEG and 60 wt.% PLA, sample A4 containing 40 wt.% PEG and 50 wt.% PLA, and sample A5 containing 60 wt.% PEG and 30 wt.% PLA.

#### 2.2.3. Preparation of CPCMs Samples

The raw materials were hand-mixed for 1 min and poured into the feeding port of a torque rheometer. The temperature of the torque rheometer was set to 185 °C and the time was set to 10 min. The material underwent a process of pressure, flipping, melting, crushing, and steady-state flow in the torque rheometer. The processed material was transferred to a flat vulcanizing machine and processed into a thin cake.

### 2.3. Characterization

#### 2.3.1. Fourier-Transform Infrared Spectroscopy (FTIR)

CPCMs were analyzed using Fourier-transform infrared spectroscopy (FTIR Nicolet IS10) by scanning the samples using a KBr pellet. The spectra were obtained in the wavenumber range of 4000–400 cm^−1^ with a resolution of 2 cm^−1^ to determine whether the compatibility between the materials was chemical or physical.

#### 2.3.2. Scanning Electron Microscopy (SEM) Analysis

The microstructure of the fracture surface of the specimens was observed by SEM (Merlin Compact, JSM-6360LV). The samples were prepared by cutting the molded specimens used for the impact strength test, and the fractured surfaces were polished to ensure that they were flat enough to be mounted on the SEM stage. The acceleration voltage (EHT) was set to 7 kV, the working distance was 8.9 mm, the magnification was 10kx, and the detector used was SE2.

#### 2.3.3. X-ray Diffraction (XRD) Analysis

The crystal structure of CPCMs was studied using X-ray diffraction (XRD Rigaku Ultima-IV). The crystal structure of the PLA-based composites was analyzed using an X-ray diffractometer. The samples were placed on the sample holder, and the voltage, current, scan angle, and scan speed were set to 40 kV, 40 mA, 5°–60°, and 2°·min^−1^, respectively.

#### 2.3.4. Nanoindentation

The mechanical properties of CPCMs were studied using a nanoindentation instrument (Agilent G200) based on the depth-sensing indentation technique. The samples, which were processed using the torque rheometer, were cut into small blocks with dimensions of approximately 10 mm, and the experiments were conducted at room temperature. Each sample was tested three times under the following conditions: a peak load of 5 mN, a Poisson’s ratio of 0.400, a peak loading time of 1.000 s, and a loading and unloading rate of 1.500 mN/s.

#### 2.3.5. Differential Scanning Calorimetry (DSC)

Differential scanning calorimetry (DSC ta250) was used to study the melting temperature, crystallization temperature, and latent heat of CPCMs. Constant temperature for 1 min to eliminate thermal history. The samples were heated from 25 °C to 200 °C at a rate of 10 K/min and then cooled from 200 °C to room temperature at a rate of 20 K/min under a purified gas flow of 100 mL/min. 

#### 2.3.6. Laser Flash Analysis (LFA)

The thermal diffusivity and specific heat capacity of CPCMs at temperatures of 50 °C, 80 °C, 100 °C, and 120 °C in a nitrogen atmosphere were measured using a laser flash analyzer (Netzsch LFA457) with the CapeL model and pulse correction method to study the thermal conductivity of CPCMs.

#### 2.3.7. Thermogravimetric Analysis (TGA)

The thermal stability of CPCMs was measured using a thermogravimetric analyzer (HTG-1) under an air atmosphere. The samples were heated from 30 °C to 500 °C at a rate of 10 °C/min to study the decomposition temperature and thermal stability of CPCMs.

## 3. Results

### 3.1. FTIR Analysis

**Figure 2:**
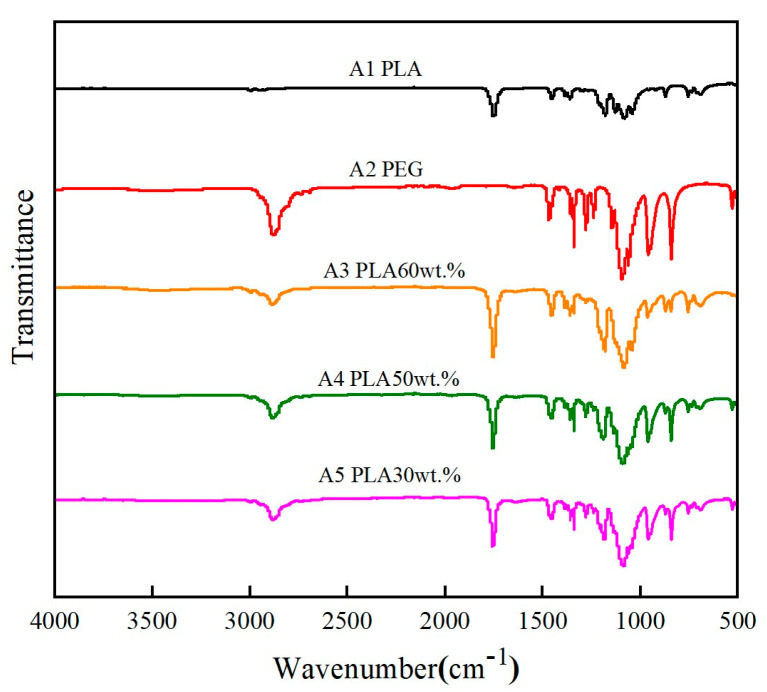
FTIR spectra of CPCMs and pure PLA.

Infrared analysis was performed on the CPCMs and pure PLA samples, as shown in Figure 2. The absorption band at 2997 cm^−1^ was assigned to the C-H stretching vibration of the CH_3_ groups in the side chains. The band at 2946 cm^−1^ was attributed to the C-H vibration in the main chain of PLA. The bands in the range of 1091–1188 cm^−1^ were assigned to the C-O-C stretching vibration of PLA and PEG. As the weight content of PEG increased from 0 to 60%, the absorption peak corresponding to the C-O-C group became stronger, which was due to the gradual increase in the C-O-C group [23]. The intensity of the C=O stretching vibration peak in the ester group of PLA at 1750 cm^−1^ increased with the addition of PEG molecules, and the peak shape became sharper [24]. The C-O stretching vibration peak at 1175 cm^−1^ for the -CH-O- group and the C-O stretching vibration peaks at 1130 cm^−1^, 1085 cm^−1^, and 1040 cm^−1^ also showed similar changes, but the positions of these peaks remained unchanged, indicating that the main chain structure of PLA remained unchanged before and after blending with PEG. These results confirmed that the prepared CPCMs exhibited non-covalent bonds [24], and the interaction forces between the two materials were manifested as physical forces [25], that is, molecular interactions or hydrogen bonding interactions.

### 3.2. SEM Analysis

**Figure 3:**
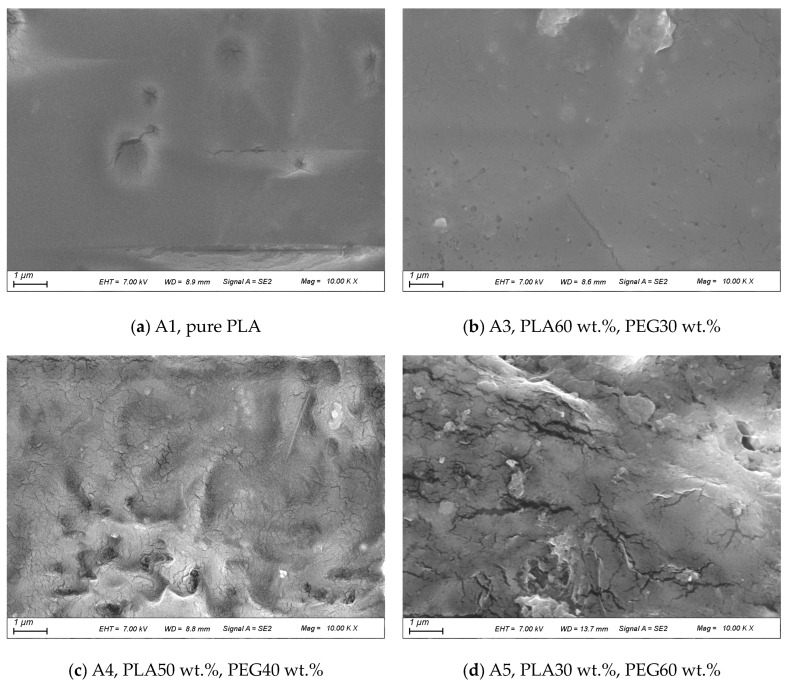
SEM images of pure PLA and CPCMs.

**Figure 4:**
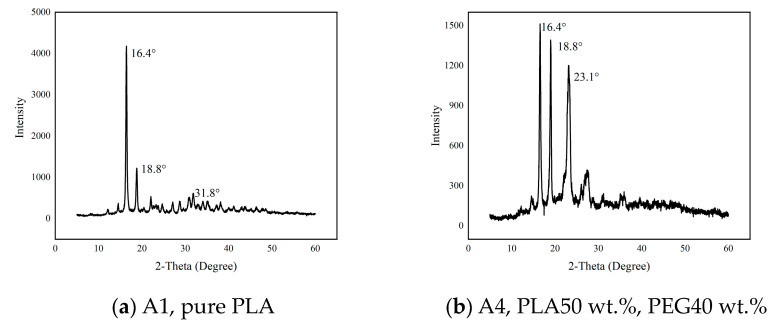
Shows the XRD patterns of pure PLA and A4 sample.

Figure 3 shows SEM images of the pure PLA and CPCMs. We observed that the surface of the pure PLA in Figure 3a was smooth and wrinkle-free, which is consistent with brittle fracture. The surface of the CPCMs containing PEG/g-C_3_N_4_ became rough with an increase in PEG/g-C_3_N_4_ content. When the PEG content increased to 30 wt.%, as shown in Figure 3b, PEG was well-dispersed in the PLA matrix without any sign of aggregation, indicating good dispersion [26]. In addition, many small voids can be seen in Figure 3b, indicating that the poor interfacial interaction load between PEG/g-C_3_N_4_ and the PLA matrix reached a critical value [27]. However, in Figure 3c and Figure 4d, there is an obvious phase separation between PLA and PEG, and in Figure 3d, 60 wt.% of the PEG is unevenly dispersed in the PLA matrix with larger structural domains, indicating poor compatibility [28]. Cracks can be seen in Figure 3c and grooves in Figure 3d, as the PEG content was too high to form uniform and dispersed structures between polymer chains due to weak intermolecular interaction forces (hydrogen bonds) [29]. In addition, white circular particles, which were PEG dispersed in the PLA matrix, are shown in Figure 3c,d [30]. Furthermore, the g-C_3_N_4_ pellets were too small to be distinguished clearly in the SEM images. Therefore, we believe that A3 (PLA: PEG: g-C_3_N_4_ of 6: 3: 1) had the best compatibility.

### 3.3. XRD Analysis

The crystallization behavior of PLA and the PLA/PEG composite materials was analyzed using an X-ray diffractometer, and the results are shown in Figure 4. As shown in Figure 4a, sharp diffraction peaks appeared near 2θ of 16.4°, 18.8°, and 31.8° for pure PLA, indicating that pure PLA crystallized under the experimental conditions. In Figure 4b, the PLA/PEG composite material with 40 wt.% added PEG showed three sharp diffraction peaks at 2θ of 16.4°, 18.8°, and 23.1°. By comparing the diffraction curves of pure PLA and pure PEG20000, it can be seen that the diffraction peak at 2θ of 16.4° belongs to the crystallization of PLA and the peak at 2θ of 23.1° belongs to the crystallization of PEG, while the diffraction peak at 2θ of 18.8° is more complex, with peaks from both PLA and PEG. At the same time, by comparing Figure 4a,b, it can also be seen that the addition of PEG changed the number of diffraction peaks of PLA, indicating that the addition of PEG disrupted the crystallization of PLA.

### 3.4. Analysis with Nanoindentation

**Figure 5:**
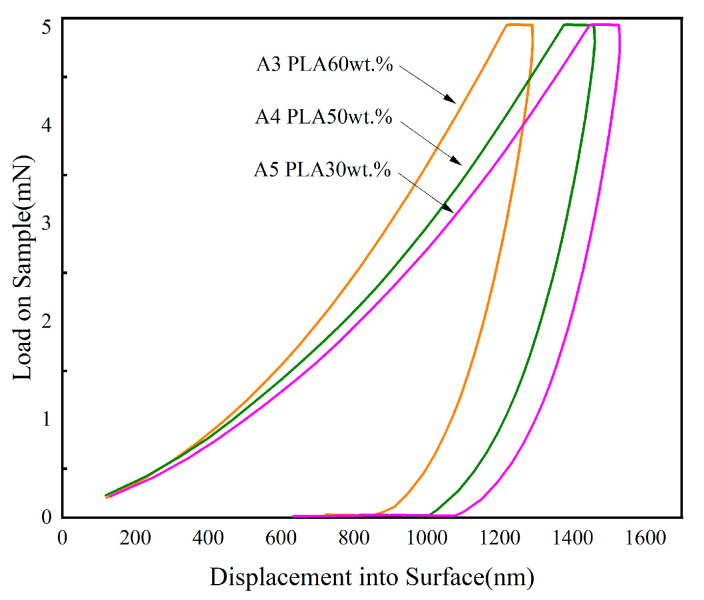
Load–displacement curve of nanoindentation experiment.

Figure 5 shows the load–displacement curves of the nanoindentation experiments, and Table 2 lists the moduli, hardnesses, and depths of the CPCMs at the maximum load under the same loading time. Obviously, as the PLA content in the CPCMs decreased, both the modulus and hardness showed a decreasing trend, while the depth showed an increasing trend. It has been reported that the modulus and hardness of pure PLA are 4.720 GPa and 0.2290 GPa, respectively, which are higher than those of the CPCMs [31], indicating that the load of PEG in the PLA composite material led to a slight decrease in hardness [32]. This is because the addition of PEG destroyed the crystallization of PLA, as analyzed by XRD and SEM. As the PEG content increased, the hardness of the CPCMs decreased. However, this compensated for the deficiency of a single PLA or PEG system as structural material, making CPCMs suitable for applications in fields such as solar thermal power generation, transportation, and construction.

### 3.5. Phase Change Temperature and Latent Heat

**Figure 6:**
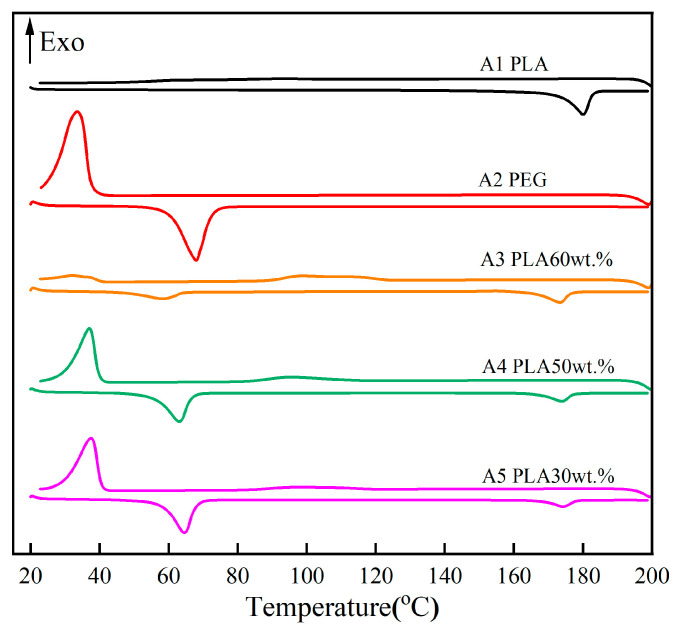
The DSC curves of the CPCMs.

The energy storage capacity of the prepared CPCMs was studied by investigating their phase transition temperatures and latent heats. Figure 6 shows the DSC curves of pure PLA, PEG, and CPCMs. The enthalpies and phase transition temperatures of samples A1–A5 are listed in Table 3. The melting temperatures of PEG and PLA were 68 °C and 180 °C, respectively. The phase transition enthalpy of pure PLA was 68.12 J/g. PLA mainly underwent a solid–solid phase change and the main transformation was a secondary transformation. The enthalpy of the phase change of pure PEG was 163.10 J/g. Sample A5 had the highest melting and crystallization enthalpies, which were 106.1 J/g and 80.05 J/g, respectively.

The melting temperature (Tm) and crystallization temperature (Tc) of pure PLA were 180.02 °C and 91.62 °C, respectively. Tm decreased by 3–4 °C for samples A3–A5 with increasing PEG content, which was believed to be caused by the phase separation of PEG in the PLA mixture [33]. The observation of the bimodal distribution of PLA/PEG also indicated no interaction between the two polymers. When the PEG content exceeded 30 wt.%, i.e., A4–A5, excess PEG aggregates, resulting in an increase in Tm.

As can be seen from the graph, pure PEG shows a distinct exothermic peak during the cooling process. PEG is a crystalline polymer and is easy to crystallize during cooling, resulting in a crystallization exothermic peak. According to Table 3, the Tm of 30 wt.% PEG (A3) was between that of pure PLA and pure PEG, with a higher heat of fusion than A4 and A5. In addition, the crystallization temperature (Tc) was lower than that of A4 and A5 because the chain mobility of 30 wt.% PEG was the strongest [34]. At the same time, the melting enthalpy of 30 wt.% PEG (A3) was the lowest, and the melting enthalpy always corresponded to the crystallization enthalpy. The crystallization enthalpy indicates the crystallinity of the process. Therefore, it was concluded that 30 wt.% PEG (A3) can effectively increase the plasticity of CPCMs [35], which is consistent with the SEM images. Compared with pure PLA, the addition of PEG lowered the melting point of the CPCMs, which was the result of PEG plasticization [36].

### 3.6. Energy Density and Thermal Conductivity

Table 4 presents the specific heats of the CPCMs at different temperatures, showing an increase in specific heat with temperature. The specific heat is a function of temperature and reflects the sensible storage capacity of the sample. According to the average value, the apparent thermal energy storage density follows the order of A4 > A3 > A5 > A1. However, considering the SEM and other analyses, A3 was found to be better than A4, and thus, A3 (PLA: PEG: g-C_3_N_4_ of 6: 3: 1) was considered the most favorable formulation.

The thermal conductivities of pure PLA and CPCMs are analyzed in Table 5, which shows a decreasing trend with increasing temperature. This trend is consistent with previous reports on CPCMs [37]. It was observed that CPCMs containing a higher percentage of PEG exhibited a higher thermal conductivity, while pure PLA exhibited the lowest thermal conductivity. Furthermore, it was found that the average thermal conductivity of pure PLA was 0.25 W/(m·K), which is consistent with the literature reported by Zhang Lai et al. [38] of 0.23 W/(m·K). A significant improvement in thermal conductivity was observed in samples A3, A4, and A5, with increases of 20%, 16%, and 28%, respectively, when compared to pure PLA. This improvement was attributed to the addition of g-C_3_N_4_ and PEG, which have higher thermal conductivities than PLA. Specifically, g-C_3_N_4_ has a thermal conductivity of 45.4 W/(m·K) and PEG has a thermal conductivity of 0.3 W/(m·K) [39]. Therefore, it was concluded that A5 and A3 were the favorable formulations.

### 3.7. Thermal Stability

**Figure 7:**
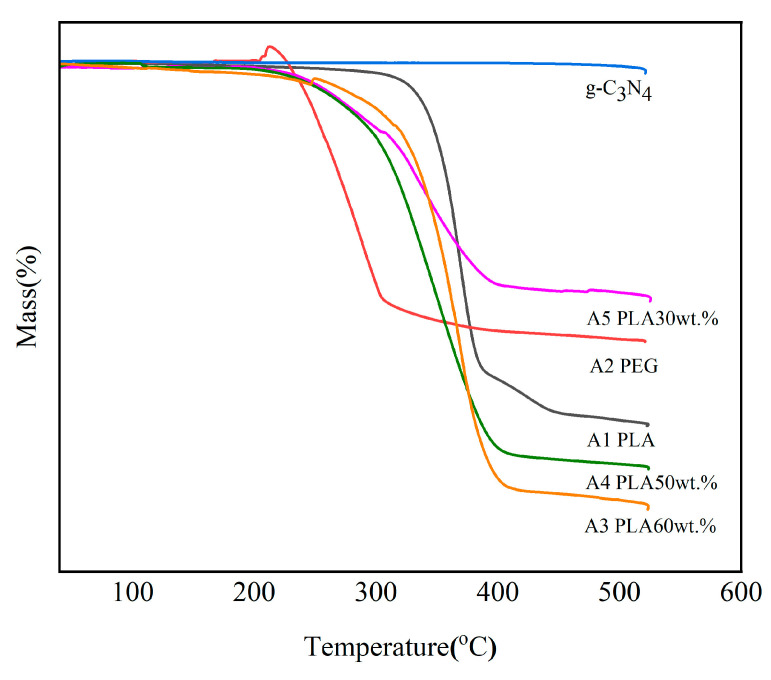
TG plot of the sample under an air atmosphere.

Thermal stability is an important factor in the study of energy storage. The thermal stabilities of CPCMs, pure PLA, PEG, and g-C_3_N_4_ were measured and studied using TG under an air environment, which is shown in Figure 7. As the temperature increased, the samples exhibited thermal decomposition, and the degradation curves only had a first-order plateau due to the chemical bond cleavage and degradation between the polymers. The temperature at which the mass loss is 5% in the TG measurement was determined as the thermal stability temperature [40]. The thermal stability temperature of the CPCMs was 241 °C, indicating that they had good thermal stability within 250 °C. When the temperature exceeded 250 °C, the polymer began to decompose, with a mass loss of about 50% observed at 250~400 °C. After 400 °C, the polymer reached a stable state and underwent cracking of the material and cleavage of the chemical bonds. In conclusion, we believe that A3, A4, and A5 meet the requirements for use at medium and low temperatures.

## 4. Energy Storage Mechanism of CPCMs

**Figure 8:**
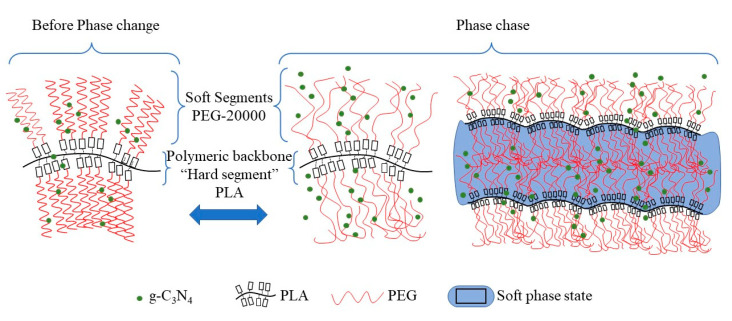
Energy storage mechanism diagram.

The energy storage mechanism is shown in Figure 8. The solid–liquid phase change material PEG20000 was modified into a solid–solid phase change material after being mixed with PLA, where the “soft phase” refers to PEG20000 and the “hard phase” refers to PLA. When the temperature reached the melting point of the soft phase, the soft phase changed from a solid to a viscous fluid state, absorbing heat in the process. PEG molecules were inserted into the PLA molecular chain, which acted as a lubricant and increases the distance between chains, thereby increasing the free volume between the materials and making the phase change of PEG more smooth, improving the efficiency of energy storage. At the same time, due to the addition of PEG20000, the viscosity of the blend system increased, causing the flowability of PLA segments to decrease. This allowed the “hard phase” to still serve as a solid support material for the entire material, ensuring the smooth absorption and release of energy by the entire material.

## 5. Conclusions

In this study, a solid–solid, bio-based, low-temperature composite phase change energy storage material (CPCM) was prepared by the torque-rheometer-mixing of ultra-high-molecular-weight PEG20000 and PLA as macromolecules, and g-C_3_N_4_ as a thermal conductivity enhancer and flame retardant. The essence of the CPCM is a solid–liquid phase change material, in which the “soft phase” is PEG20000 and the “hard phase” is PLA. When the temperature reaches the soft phase melting point, the hard phase serves as a skeleton to maintain the stability of the material, and the soft phase changes from solid to Viscous fluid state to absorb heat. PEG molecules are inserted into the PLA molecular chain, making the PEG phase transition smoother and improving the efficiency of energy storage. Whether the CPCM was physically blended or chemically cross-linked was characterized by FTIR. SEM showed that when the PLA: PEG: g-C_3_N_4_ ratio was 6: 3: 1, the compatibility critical value was reached, and phase separation occurred when the PLA content was reduced. XRD indicated that the addition of PEG destroyed the crystallinity of PLA, resulting in a decrease in material hardness, which is consistent with the results of the nanoindentation testing. In terms of thermal properties, the CPCM exhibited high enthalpy (106.1 kJ/kg), good thermal conductivity (0.32 W/(m·K)), and excellent thermal stability, and the material remained stable below 250 °C. Moreover, the addition of g-C_3_N_4_ endowed CPCM with flame retardancy. In summary, when PLA: PEG: g-C_3_N_4_ ratio was 6: 3: 1, it had the best overall performance. Therefore, we think it can be applied to the construction field for sandwiching fireproof materials, or biodegradable solar power panels, etc.

## Figures and Tables

**Table 1:** Formulation of composite phase change energy storage materials (CPCMs).

Sample	A1	A2	A3	A4	A5
PEG (wt.%)	0	100	30	40	60
PLA (wt.%)	100	0	60	50	30
g-C_3_N_4_ (wt.%)	0	0	10	10	10

**Table 2:** Modulus, hardness, and depth at maximum load of CPCMs.

Sample	A3	A4	A5
Modulus At Max Load (GPa)	3.577	3.267	3.044
Hardness At Max Load (GPa)	0.137	0.114	0.096
Depth at Max Load (nm)	1291.345	1491.341	1531.124

**Table 3:** Phase transition temperatures of CPCMs.

	**Melt**						
**Sample**	**Fusion** **Temperature** **Tmo1 (°C)**	**Melting Point** **Tmp1 (°C)**	**Latent Heat** **ΔHm1 (J/g)**	**Fusion** **Temperature** **Tmo2 (°C)**	**Melting Point** **Tmp2 (°C)**	**Latent Heat** **ΔHm2 (J/g)**	**Total Latent Heat Total** **ΔHm (J/g)**
A1	/	/	/	173.65	180.02	68.12	68.12
A2	60.25	68.00	163.10	/	/	/	163.10
A3	47.28	58.36	30.99	165.94	173.29	27.81	58.80
A4	56.02	63.06	83.69	167.14	173.83	21.19	104.88
A5	58.29	64.54	89.80	168.40	174.15	16.30	106.1
	**Crystallization**						
**Sample**	**Crystallization Temperature** **Tco1 (°C)**	**Tcp1 (°C)**	**Latent Heat** **ΔHc1 (J/g)**	**Crystallization Temperature** **Tco2 (°C)**	**Tcp2 (°C)**	**Latent Heat** **ΔHc2 (J/g)**	**Total Latent Heat Total** **ΔHc (J/g)**
A1	/	/	/	105.08	91.62	3.27	3.27
A2	37.35	33.57	114.89	/	/	/	114.89
A3	39.90	32.23	7.42	123.55	98.27	22.10	29.52
A4	39.73	36.97	64.85	114.90	94.63	15.01	79.86
A5	40.41	37.51	68.73	119.15	96.43	11.32	80.05

Tmo1 indicates the melting point onset temperature of the first peak; Tmp1 indicates the peak melting point temperature of the first peak; Tmo2 indicates the melting point onset temperature of the 2nd peak; Tmp2 indicates the peak melting point temperature of the 2nd peak; ΔHm1 indicates the enthalpy of the 1st peak; ΔHm2 indicates the enthalpy of the 2nd peak; ΔHm = ΔHm1 + ΔHm2 (heat-absorbing melt peak, lower temperature for PEG melt, higher temperature for PLA melt). Tco1 represents the starting temperature of the crystallization point of the first peak; Tcp1 represents the peak temperature of the crystallization point of the first peak; Tco2 represents the starting temperature of the crystallization point of the second peak; T_C_p2 represents the peak temperature of the crystallization point of the second peak. ΔHc1 represents the enthalpy value of the first peak; ΔHc2 represents the enthalpy value of the second peak; ΔHc = ΔHc1 + ΔHc2 (exothermic melting peak, the lower temperature corresponds to PEG crystallization, and the higher temperature corresponds to PLA crystallization).

**Table 4:** Specific heat of CPCMs at different temperatures.

T (°C)	30	50	80	100	120	Average
A1	1.87	2.01	2.21	2.32	2.36	2.15
A3	1.79	3.91	2.17	2.20	2.24	2.46
A4	2.02	3.18	2.51	2.52	2.60	2.57
A5	1.74	2.65	2.29	2.24	2.29	2.24

**Table 5:** Thermal conductivity and enhancement.

T (°C)	50	80	100	120	Average	Enhancement
A1	0.28	0.26	0.23	0.21	0.25	0
A3	0.54	0.25	0.22	0.19	0.30	20%
A4	0.40	0.29	0.26	0.24	0.29	16%
A5	0.43	0.33	0.28	0.26	0.32	28%

## Data Availability

The data presented in this study are available on request from the corresponding author.
